# Copilot in service: Exploring the potential of the large language model-based chatbots for fostering evaluation culture in preventing and countering violent extremism

**DOI:** 10.12688/openreseurope.19612.2

**Published:** 2025-04-17

**Authors:** Irina van der Vet, Leena Malkki

**Affiliations:** 1 Centre for European Studies, University of Helsinki Faculty of Social Sciences, Helsinki, Uusimaa, 00014, Finland; 2 Vrije Universiteit Brussel Institute for European Studies, Brussels, Brussels, 1050, Belgium; 3 Centre for European Studies, University of Helsinki Faculty of Social Sciences, Helsinki, Uusimaa, 00014, Finland

**Keywords:** Artificial intelligence (AI), large language model (LLM), recommender system, evidence-based evaluation, evaluation culture, and preventing and countering violent extremism (P/CVE).

## Abstract

**Background:**

The rapid advancement in artificial intelligence (AI) technology has introduced the large language model (LLM)-based assistants or chatbots. To fully unlock the potential of this technology for the preventing and countering violent extremism (P/CVE) field, more research is needed. This paper examines the feasibility of using chatbots as recommender systems to respond to practitioners’ needs in evaluation, increase their knowledge about the key evaluation aspects, and provide practical guidance and professional support for the evaluation process. At the same time, the paper provides an overview of the limitations that such solution entails.

**Methods:**

To explore the performance of the LLM-based chatbots we chose a publicly available AI assistant called Copilot as an example. We conducted a qualitative analysis of its responses to 50 pre-designed prompts of various types. The study was driven by the analysis questions established to explore accuracy and reliability, relevance and integrity, as well as readability and comprehensiveness of the responses. We derived the key aspects of evidence-based evaluation along with practitioners’ needs from the results of the H2020 INDEED project.

**Results:**

Our findings indicate that Copilot demonstrated significant proficiency in addressing issues related to evidence-based evaluation in P/CVE. Most generated responses were factually accurate, relevant, and structurally sound, i.e. sufficient to kick-start and deepen internal evidence-based practise. At the same time, biases and data security issues inherent in LLM-based chatbots should be carefully explored by practitioners.

**Conclusions:**

This study underscored both the potential and limitations of LLM-based chatbots in fostering evaluation culture in P/CVE. While Copilot can effectively generate accessible, informative and encouraging recommendations, it still requires a professional oversight to manage and coordinate the evaluation process, as well as address more field-specific needs. The future research should focus on more rigorous and user-centred assessment of such systems for P/CVE use based on multidisciplinary efforts.

## Introduction

The advancement of artificial intelligence (AI) and, specifically, generative AI has impacted all sectors of our lives. AI-based technologies have integrated into everyday life through innovations such as chatbots, virtual assistants, voice recognition systems, or image generators. However, it is still often misunderstood what AI acctually means. In general terms, AI is seen as a system that simulates human behavior to perform tasks requiring intelligence ( Banh & Strobel, 2023; Ooi *et al.*, 2023). The European Union's AI Act defines an AI system as “a machine-based system designed to operate with varying levels of autonomy, that may exhibit adaptiveness after deployment and that, for explicit or implicit objectives, infers, from the input it receives, how to generate outputs such as predictions, content, recommendations, or decisions that can influence physical or virtual environments" ( EU 2024/1689). This definition particularly highlights the operational mechanism of these systems and the far-reaching impacts they can have on both digital and real-world contexts.

Natural language processing (NLP) is a critical component for grasping the current development of generative AI, as it enables human-like language comprehension and generation. This includes understanding, interpreting, and producing human-written or spoken language, as well as discerning context within interactions ( Segura, 2023). NLP have been significantly improved in the recent years due to the large language models (LLMs), a type of generative AI that is built upon statistical algorithms and trained on extensive datasets from diverse sources for generating human-like output ( Routray *et al.*, 2023).

Since the release of Open AI’s openly available ChatGPT in November 2022, the potential uses of LLM-based chatbots ^1^ – part of broader generative AI – have been explored extensively in various diciplines. This trend includes the field of preventing and countering violent extremism (P/CVE). So far, discussions in this field have focused primarily on two areas: how these chatbots can be, or are already being, used by individuals or groups that support violent extremism, and how they can be useful for detecting and moderating violent extremist content online.

This article introduces a new, yet unexplored, perspective on using chatbots to advance evidence-based policy and practice in P/CVE. Specifically, it examines the possibility of employing chatbots to assist policymakers and practitioners in building or enhancing their professional capacity and knowledge about P/CVE. Although chatbots have been used for educational purposes in other contexts, to our knowledge, they have not yet been utilised in this way within the P/CVE field.

The article centres on one key element of evidence-based policy and practice – evidence-based evaluation. We conduct a preliminary qualitative study to understand what Copilot, as one of the publicly available chatbots, can say about evaluation in the context of P/CVE and what recommendations it can provide for professionals engaging in a conversation with it. Public chatbots might have a number of limitations compared to domain-specific ones, but they can still demonstrate the potential of this technology for P/CVE. The purpose is thereby to explore how these public chatbots can be used to acquire knowledge about evaluation and handle evaluation tasks in P/CVE, outlining both the benefits and drawbacks of such systems for relevant practitioners.

The article will proceed as follows. We begin by describing the state of evidence-based evaluation in the P/CVE field and how a chatbot could contribute to advancing both the culture and practice of such evaluations. Next, we provide a brief review of research on the impact and potential use of LLM-based chatbots for P/CVE work. Following this, we discuss the latest advancements in chatbot development, particularly their role as recommender systems, drawing from research results about similar uses of chatbots in other fields to set realistic expectations based on previous experiences. In the next part of the article, we present an analytical review of our dataset consisting of 50 relevant prompts and Copilot’s responses. We explain our methodology for exploring accuracy and reliability, relevance and integrity, as well as readability and comprehensibility in Copilot answers regarding evidence-based evaluation in P/CVE. Importantly, we also describe the limitations of this study and formulate the suggestions for further research as part of our methodology.

Finally, we discuss the potential benefits and challenges associated with utilising chatbots for capacity-building and strengthening an evidence-based approach in the P/CVE field. While the primary focus of the article is on evidence-based evaluation, the findings can be valuable for mapping broader possibilities for using chatbots in counselling or trainings in the P/CVE field.

### State of art in evaluations and evaluation culture in P/CVE field and potential contribution of LLM-based chatbots

The origins and motivation for this study derive from the well-documented lack of evidence-based evaluations in the P/CVE field ( Silva & Kordaczuk-Wąs, 2025) and analyses on what can be done to improve the situation. P/CVE is a relatively new field which is, in many respects, still taking shape. There are currently no established standards or models for how P/CVE initiatives could or should be evaluated. Evaluations remain rather uncommon, and their results are often not widely shared. At the same time, there is a broad consensus that more evaluations are needed. Evaluation is a key part of evidence-based policymaking and practice, as it provides an opportunity for P/CVE stakeholders to assess their own programmes and policies critically, learn from the findings, and utilise the results to improve the effectiveness of their work. Cumulatively, evaluations help establish what kind of policies and programmes work to prevent and counter violent extremism and under what conditions.

Furthermore, previous research and experience has shown that a key to successful and productive evaluations is to consider them a systemic part of organisational processes (Ibid.). Instead of separate, one-time events, they should be seen as an integral part of the programme development and implementation cycle. Ideally, evaluations are supported by evaluation culture, which is characterised by a commitment to utilise evaluation as an instrument for learning and an active search for such evidence to further improve the processes and practices. This also means that evaluation is not only something that an evaluator does. Successful planning, implementation and utilisation of evaluations also requires good knowledge of the basic principles and practices of evaluation from those who plan and implement programmes and policies. This is because successful evaluation depends on good advance planning to ensure that all data and documentation needed for the eventual evaluation are collected and produced at different points of the implementation.

One initiative to improve the current state of affairs in the P/CVE field was the Horizon 2020 funded INDEED project (2021–2024), which engaged a wide group of European P/CVE stakeholders representing law enforcement, the prison sector, non-governmental organisations, policymakers, and academic researchers in finding ways to advance evidence-based evaluation in various segments of P/CVE. The gap analysis at the beginning of the project, as well as several workshops throughout its duration, provided helpful insights into why evaluations have not been more common in the field. There are several reasons for this, for example, a lack of resources and funding, lack of trust towards evaluators, and the fear that the results of the evaluation may have negative consequences for the programme or policy. Most notably for this current study, P/CVE practitioners and policymakers repeatedly mentioned lack of knowledge and expertise on evaluation as one major obstacle. They would like to engage in evaluation, but do not quite know how and where to begin. This is a critical development need, as high-quality evaluations will not be possible without practitioners and policymakers who know how to plan and implement programmes and policies with evaluation in mind.

Numerous tools, resource banks, and guidebooks have been produced to advance P/CVE policymakers’ and practitioners’ awareness and knowledge about evaluation, among them the IMPACT Europe Evaluation toolkit for professionals working in the counter violent extremism field ( IMPACT Europe), the INDEED project’s evidence-based evaluation package ( INDEED), or the USAID CVE reference guide for local organisations ( USAID CVE). These typically take the form of web-based tools, PDFs, and resource lists that summarise the key information about evaluation for P/CVE practitioners and policymakers and help them find further information on specific questions. These resources are certainly helpful for gaining a basic understanding of evaluation principles and processes, but they do not fully meet the diverse evaluation needs of P/CVE professionals operating in fast-paced and rapidly changing environments. P/CVE professionals, while appreciating the general-level knowledge, have also communicated that they need more tailored information about evaluations and more examples, depending on the type of initiative, context, objectives, or target groups. This is something that the current knowledge resources cannot provide in a compact way. What makes such specified information all the more relevant is that P/CVE programmes and policies often have significant political, ethical, and social sensitivities. They may involve the participation of (or otherwise affect) vulnerable populations, collect and use sensitive data, and generate responses and effects in the wider communities. These sensitivities vary from one programme or context to another and affect the possibilities and conditions of evaluation. P/CVE professionals already consider the current solutions quite cumbersome to use and navigate, even when they are well designed, so simply adding more information to the existing tools and resource banks is not a feasible option. Another drawback of the current resources is that they age and require a lot of resources to sustain and update them. This work will multiply if the resources are to be maintained in several languages.

To meet the P/CVE professionals’ needs, it would be helpful to come up with new tools that could provide more specific information and do it in a way that would not require the professional to spend a lot of time looking for the relevant information on a website or guidebook. The LLM-based chatbots could potentially serve as an effective tool or an overall solution to meet these needs. By inputting a prompt in the format of a question, scenario, or task in the chatbot at their most convenient time and device (computer or a mobile telephone), professionals would ideally receive a response that speaks directly to their situation. Through the natural conversation, they could consult on their problems, receive advice, and even be emotionally encouraged in their work. This would require the chatbot to “know” enough about evidence-based evaluation and P/CVE and “understand” who the user is, as well as to produce reliable, well-organised, and comprehensive information. At the same time, when relying on such solutions, it is also important to know all their limitations.

### LLMs and research on their use in the P/CVE field

Before exploring the LLM chatbot Copilot, it is worth saying a few more words about what LLMs are and going through previous studies on their relevance and application to P/CVE.

LLMs are advanced AI systems capable of processing and understanding human language through interactions, allowing them to engage in conversations that simulate human dialogue ( Mohamed & Kumar, 2024). The LLM functions by “feeding” an AI with a large amount of existing digital data (mainly from internet sources), which then allows it to understand human language and respond to prompts (questions and tasks) by generating new text or an image based on the inputted and disposed data or a description ( Zhao *et al.*, 2023). Most recent models are *generative pre-trained transformer* (GPT) models ( Carugati, 2023). They can serve as the basis for many tools that require Natural Language Processing (NLP). OpenAI Inc. released a publicly available ChatGPT in November 2022 and currently GPT is widely used as a “foundation model” for creating cost-effective AI innovations (Ibid.). The model can be used for generating “text, image, video, code, sound, and other produced content” ( Banh & Strobel, 2023) in multidisciplinary and multimodal domains.

To date, the most popular generative AI chatbots include ChatGPT ^2^, Google’s Gemini (previously called Bard), chatbot Claude, built by Anthropic, and Microsoft Copilot, but more and more solutions have appeared in the market recently. Even though all the chatbots are different and there are different opinions about their usefulness for various use cases, they are still gaining popularity among both the general population and professionals. While chatbots’ usability in different tasks has been actively studied over the last two years, more multidisciplinary research across different fields is needed to analyse the capabilities and potential functions of such systems and extract the lessons learnt.

The potential and actual uses of chatbots (and generative AI in general) have also been studied in the P/CVE field. The research so far has centred mainly around two themes. The first group of studies speaks about the capability of LLM-based chatbots to detect and track radical content and misinformation online. The second group of studies focuses on misuse cases of such systems for generating radical content and misinformation, which can (potentially and more realistically) increase the risk of escalation in violent extremism.

*The first group* includes studies that explore the potential of LLMs for detection of extremist propaganda and terrorism, for example the study by Dong *et al.* (2024), in which they use LLM (GPT3.5 and GPT4) to detect and classify (into extremist and non-extremist) metacontextual online extremist posts on social media. They use prompt engineering to evaluate the performance of an LLM in labelling the content. Prompt engineering “refers to the process of designing and refining input instructions to effectively guide LLMs in performing specific tasks” (Ibid., p. 14). The authors concluded that the better the prompt is formulated and the task is described, the better the LLM defines extremist content.

Another similar study is that of Shah *et al.* (2024), who transformed and finetuned the raw LLM architecture called DistilRoBERTa into a cost-effective tool for detecting terrorism through classification and text analysis. The model was trained with curated data. Although the results of the study did not discuss the effectiveness of LLMs for detecting terrorism-related propaganda directly, they still demonstrate that LLM show great potential in classifying terrorism-related and offensive texts. What still has to be done to increase the relevance of this approach is to detect all potential biases and build mitigation measures well in advance, for example in reducing false positives or increasing context understanding.

Another team of scholars from this group, Szwoch *et al.* (2024) have also assessed the usefulness and helpfulness of pre-trained LLMs (GPT) in detecting online propaganda in terms of cost effectiveness and time efficiency in elicitation of harmful content. They conducted the prompt-driven experiments with different datasets in English and Polish, using base prompts (statements/questions that required just the answer) and chain of thought (a series of follow-up questions, enabling the LLM to show the reasoning behind the given answer). Both prompts featured examples of each of the propaganda techniques by utilising a few-shot approach that centred on several prompts or queries to guide a chatbot towards expected responses (Ibid.). The generated outcomes of the LLM were evaluated with the help of SemEval2020, a semantic evaluation program. Although the study demonstrated that the LLMs were generally not as effective in propaganda detection as expected, the idea of further advancement of LLMs and the methods for their evaluation still brings a lot of hope for this use case.

At the same time, there are related studies in other disciplines that prove the general ability of LLM-based chatbots to detect propaganda. Li *et al.* (2024) explored the role of ChatGPT in detection of harmful, hateful, toxic, and discriminatory content in social media with a remarkable accuracy of 80%. This result initially demonstrates the transferable value of this approach to P/CVE, even though more elaborated testing and evaluation are needed to prove the effectiveness of this approach for the sector.

*The second group* of studies explores the potential misuse of LLM-chatbots by extremist groups. Many scholarly discussions between 2020 and late 2023 were mostly hypothetical warnings about the misuse of AI in general, and LLMs and chatbots (eg., Lakomy, 2023, or EUROPOL, 2023) in particular, to purposefully fuel radicalisation, spread propaganda and commit extremism-related crime. One of the reasons behind this is the acknowledged securitisation of AI ^3^. These systems have been associated with risks mainly because it is possible to customise these models to generate any desired content more efficiently. McGuffie & Newhouse (2020) argue that GPT-3 API could be “weaponized” by extremists who could use it for spreading their ideologies and recruiting new members. The authors then utilise trained machines based on GPT2 and GPT3 models for their experiment with inputting different prompts. The data for training included a dataset of extremist manifestos and a wide range of data of different styles, covering different spectrums of extremism. The results demonstrated the ability of both systems to generate content that can potentially lead to online recruitment. The authors indicated the need for policymakers and all of society to define social and political mechanisms against “machine-generated disinformation and propaganda” (Ibid., p. 1).

It is important to note that it is challenging to establish the fact and collect evidence that these technologies are actually used by radicalised and criminal groups because it requires specific infrastructure, as well as a set of appropriate skills, methods, and expertise. However, in the past two years or so more evidence has begun to arrive from research and practice about actual cases where generative AI and LLM systems have been misused. Nelu (2024) notes that terrorist organisations (eg. Islamic State) have started producing guidelines about how to use generative AI technologies to spread misinformation and propaganda, which work as tutorials for their followers. The authors pointed out that AI chatbots in particular could be used effectively for recruiting and indoctrination by building human-like personal relationships (ibid.). Another paper ( Allchorn, 2024) explored the online posts of far-right extremists in the US, UK, Australia, and New Zealand. The paper discussed how extremist groups within the global far-right used AI methods for their own purposes. The author conducted an analysis of content from 12 violent and non-violent far-right groups, finding that they were exploring ways to utilise AI to stay ahead of counter-terrorism efforts. For example, AI’s LLMs themselves, as has been shown, were misused to spread violent ideas for “recruitment and kinetic attacks” (Ibid., p.13). The paper also mentioned that these far-right groups are concerned about the alleged liberal bias in current AI models and are seeking alternative models that align with their own libertarian or conservative values. They propose using clones of popular AI models such as ChatGPT (eg. RightWing, Freedom & Truth GPT and HuggingFace), which have been tailored to promote right-wing agendas.

### Pros and cons of using LLM chatbots as a recommender system

While the two groups of studies mentioned previously are prominent in the research arena, a third emerging group explores LLM’s full linguistic potential, including its application in providing recommendations, explanations, and guidance – as seen in conversational chatbots. Lloyd (2023) describes chatbots’ speech as “chatsplaining”, similar to the word “mansplaining”, when a chatbot explains something in a patronising and very detailed manner. “Speaking” in such a manner, the chatbot reveals its potential to act not only as a professional companion but also a mentor who supports and provides useful recommendations. Having said that, this part specifically examines the use of LLMs as recommender systems to support P/CVE professionals in their work. For this purpose, it is essential to underline key challenges, possibilities, and limitations associated with using LLMs in this context. Notwithstanding their limited use in P/CVE so far, there are studies exploring similar applications in other fields.

So what are those recommender systems and where do they come from? The principles underlying recommenders have evolved over the past century. Dating back to the 1970s, cognitive scientists developed the first relatively primitive computer-based librarian, named Grundy, who suggested literature to readers by classifying them into specific groups upon their preferences ( Ekstrand *et al.*, 2011; Rich, 1979). By “learning” about its users and offering its services accordingly, the early system demonstrated a rudimentary form of the current recommender system. Today, recommender systems are online tools that filter and personalise information to save users’ time when searching for relevant data ( Roy & Dutta, 2022). A recommender system is an information-filtering tool that suggests items (generally, movies, products, or music) to users based on their preferences and behaviour. It uses algorithms to analyse user data and provide personalised recommendations.

Recommender systems are closely linked to AI as they often make predictions about users’ interests to offer relevant suggestions ( Zhang *et al.*, 2021). The incorporation of AI enables recommender systems to learn from user interactions, adapt to changing preferences, and provide more accurate recommendations over time.

As Lloyd (2023) notes, despite initial perception of chatbots as “random arrangements of letters floating in alphabet soup” from a technological perspective, what they produce does indeed make sense (p.5). Hence, the recommendations produced by chatbots could potentially sound valuable. However, one significant limitation of LLM chatbots is their inability to generate content that is 100% factually reliable. Human effort is often still required to verify the accuracy of the information provided, particularly when using chatbots for professional purposes. It is worth to mention here that AI-based technologies are rapidly improving in terms of accuracy. Tech companies have been mitigating “bullshitting” and hallucinations that have been of a great concern since the early development of AI, by taking mitigation and optimisation measures ( Lei *et al.*, 2025; Zhao *et al.*, 2023). However, the issue is far from being entirely resolved and LLMs in general still hallucinate and produce inaccurate content. The answers produced by chatbots still pose some challenges, especially when trusted by humans ( Steyvers *et al.*, 2025) and this is especially evident in educational contexts where students expect to find rich information and use them explicitely for learning ( Rudolph *et al.*, 2023).

Since 2020 there has been an increasing number of studies on how to govern the interaction between humans and machines. A relatively new concept, “human-in-the-loop”, highlights the crucial role of humans in controlling AI output and assessing its usability ( Mosqueira-Rey *et al.*, 2023; Xu, 2019). We argue that another important consideration is the need for a “professional-in-the-loop”, who not only applies common sense to AI-generated content but also brings solid knowledge and expertise to verifying accuracy and informing decision-making based on independent judgements. 

The current recommender systems are largely advanced by LLMs. Furthermore, LLM-based recommendations produced by chatbots have shown promising results in the accuracy, relevance, and usability of provided recommendations (see Dehbozorgi *et al.*, 2024), when based on Retrieval Augmented Generation (RAG). RAG is a working principle of LLMs that enhances their reliability through the utilisation of an external knowledge base ( Gao *et al.*, 2023). The advantage of RAG is that it reduces the chances of receiving irrelevant results in response to one’s query. However, the answers produced might still not be 100% accurate (Ibid). This principle holds promise when implemented for chatbots. Complementing an LLM with a reliable local dataset can lead to more rigorous and nuanced responses in relation to professional fields via the chat. Overall, it seems realistic to expect that AI chatbots will function increasingly independently and with fewer factual errors and hallucinations in the near future.

There are also several ethical questions that need to be kept in mind when using LLM-based chatbots (see Chow & Li, 2024). When LLMs “learn” from the training data, they also learn the biases inherent in that data. In the case of RAGs, when the data from the knowledge base is polluted with biases, the results could be consequential. Gover (2023) exemplifies various biases that LLM models produces, such as gender, racial, or religious biases. It is therefore important to consider how to detect and correct these actual and potential biases so that they are not reflected in the recommendations provided by LLM chatbots.

Another question relates to privacy and data security. Even if developers claim that LLMs are not trained with personal information, users might still include personal or sensitive data in the queries. It becomes important to define how users’ data is secured from unauthorised access ( Piñeiro-Martín *et al.*, 2023). Furthermore, it is not always clear what happens to inputted data, where it is stored, and how it is used. There is a common explanation in the terms of use or terms and conditions that the data inputted by users is used for further training or finetuning of AI chatbots, but in reality this is difficult to check. Such practices often depend on corporate ethics, which can take a back seat to the desire for customisation and profitable solutions. It is crucial to inform those who entirely rely on technology systems about the importance of not only reading the terms and conditions carefully to understand how their inputted data is used but also critically evaluating any new AI-based services before using them. This emphasis becomes particularly important for P/CVE practitioners, who often manage a lot of sensitive and private data.

A whole host of new questions emerges when the AI systems are used to make decisions that have an impact on people’s lives or when these systems operate without significant human involvement. ^4^

Finally, there are open questions related to the legal basis and regulation of LLMs, especially concerning copyright. Most openly available LLMs are trained on copyrighted material, and only in some cases, they may use direct quotations from such material in their responses to queries. There are several ongoing litigations about how copyrighted material can be legally used in this context (see eg., Ghaswalla, 2023; Gynbaum & Mac, 2023). At this point, it is important for users to be aware that publicly available LLM chatbots might produce direct quotations from the training data ( Karamolegkou *et al.*, 2023).

The AI act adopted by the European Union in June 2024 represents a significant step towards AI regulation ( EU 2024/1689). This regulatory framework also applies to general-purpose AI models, which include many LLM chatbots. The AI Act sets new transparency requirements for providers of such models and mandates compliance with the EU copyright law (Ibid.). While this does not immediately solve all challenges related to training data, it should make it somewhat easier for users to assess the lawfulness of LLMs.

## Method

### a. Our approach

The purpose of this study is to explore the usability of LLM-based chatbots for advising P/CVE professionals. To achieve this, we will focus on the potential of these chatbots in increasing knowledge on evidence-based evaluation (as one identified need in the P/CVE field) by using the Copilot chatbot ^5^.

We have chosen to utilise the Copilot chatbot by Microsoft (version from January 2025) because it has been already used in classrooms and corporate settings rather successfully; and because it is openly available, and displays all the functionalities of similar LLM-based chatbots. Additionally, Copilot is now integrated into Microsoft Office’s 365 applications, making it more functional for professional use. As the Microsoft website ^6^ promises, “Copilot, your AI companion, is ready to support you whenever and wherever you need it”. Our task in this part is to explore whether it may be true.

Copilot functions similarly to ChatGPT but with some key distinctions. It is powered by GPT-4 ^7^ ( Chin, 2023) and Bing search. This setup aligns with the RAG principle, which enables Copilot to provide references to sources, potentially increasing its reliability, usefulness, and transparency. Marais *et al.* (2024) highlight that this feature is particularly advantageous for students as it allows them to verify “the credibility of the sources from which Copilot pulled information” (p. 2). These features are crucial when using Copilot to inform professionals. However, it is also important to recognise that Copilot also displays the limitations of LLM-based chatbots in general ( Aaron *et al.*, 2024).

The key question in this study is what Copilot currently “knows” about evidence-based evaluation and how it can perform, given the specifications of the P/CVE field. To be useful to professionals, the chatbot should respond readily to the questions and queries prompted by professionals and provide reliable, relevant, comprehensive replies that are also easy to understand. 

Several methodologies have been used to assess the performance of LLM chatbots, depending on exactly what is being evaluated. The aforementioned studies assessing chatbots’ capability to provide reliable and comprehensive medical information typically use base prompts (asking separate questions). The answers have then been evaluated for their reliability and comprehensibility. Sometimes these answers were compared to the same questions answered by professionals or information provided by patient organisations. Another design involved asking professionals to distinguish between responses from the chatbot and those given by someone knowledgeable about the topic.

In this study, we adopt a user-centred and qualitative approach to explore the potential of the chatbot for P/CVE professionals. Specifically, we simulate how these professionals would use the chatbot when seeking information about evidence-based evaluation, focusing on prompts that someone with little prior knowledge might use to gain an initial understanding of the evaluation principles and procedures. These include learning how to plan evaluations, how they are conducted, and what considerations are specific to their cases. To achieve these goals, we, the authors of this paper, as domain experts, engaged in a series of dialogues with the chatbot, observing what kind of knowledge it is able to provide. Similar qualitative, dialogue-based designs have been used to explore whether and what kinds of extremist material can be produced with the help of LLMs (See eg., McGuffie & Newhouse, 2020). We designed and inputted 50 prompts in January 2025 ^8^. Ein-Dor *et al.* (2024) note that to “maximize the capabilities of LLMs, crafting effective prompts is essential. The prompts should clearly define the task and desired outcomes, conveying nuances, preferences, and important output requirements” (p. 1). Some medical studies have also favoured well-formulated prompts (see eg., Meskó, 2023). Warren *et al.* (2024) compared prompted and unprompted questions and concluded that prompted questions lead to better quality results.

Our study consists of two stages. In the first stage, we inputted 40 pre-defined prompts (Discussion 1, Box 1). Since a commonly applied typology for engineering prompts in similar social sciences reviews does not yet exist, we propose our own while drawing on some previous work. Firstly, we classified prompts by purpose of entry, as our study simulates various situations in which professionals seek information, recommendations, specific tasks through requested actions, and even emotional support. Thus we can define four categories:

**Box 1. List of prompts for Discussion 1 with Copilot**

1.     What is evidence-based evaluation and how does it apply to P/CVE?

2.     Please tell me about the methods that are used in such evaluation.

3.     Can you provide any tips on how to use these methods without previous experience in evaluation?

4.     Is using interviews enough? What is your opinion?

5.     What methods can I use for data analysis and what methods for data collection?

6.     Do I need to develop any indicators? I do not know how to do it.

7.     What are the most common types of evaluation in P/CVE?

8.     What is the difference between impact evaluation and impact assessment? And what is the difference between impact evaluation and outcome evaluation?

9.     Can you tell me more about process evaluation?

10.   If my programme has recently finished, does it still make sense to do the process evaluation?

11.   When do I need formative evaluation?

12.   I would like to do an evaluation, but I do not know what method to choose. Can you help?

13.   What exactly is evidence? How can I collect it?

14.   How do I ensure that evidence is reliable?

15.   How do I plan and implement evaluation in P/CVE?

16.   Can you visualise this pathway for me?

    a. What specific visual aid do you mean?

17.   Can you help me to get started? I am lost.

18.   Can you guide me through the theory of change? What is it and how to design it? I have no previous experience.

19.   Do you have another example of the theory of change in P/CVE? If possible, please provide the links to these examples if there are any.

20.   Can you explain each of the components of the theory in more detail?

21.   How about ethics in evaluation? Any tips and recommendations here?

22.   What about gender?

23.   And human rights?

24.   I have some sensitive data, how can I manage it?

25.   Any recommendation about how to include vulnerable groups in evaluation?

26.   How can I prepare our team for evaluation?

27.   Shall I inform funders about evaluation? Do I need to inform any organisation?

28.   I am unsure what is best to have, an internal or external evaluator?

29.   What evaluation is less biased?

30.   What is a good evaluation and what is a bad evaluation?

31.   How to make a good quality evaluation?

32.   Is there anyone who can help me with my evaluation?

33.   I need more examples of evaluation in P/CVE, can you provide them?

34.   How can I increase my skills in evaluation?

35.   Can you recommend any training in evaluation for P/CVE professionals?

36.   Do you know of any evaluation tools for P/CVE?

37.   Can you explain the advantages and disadvantages (limitations) of each of them?

38.   Do you know any evaluation models? Can you visualise them or send the link to those?

39.   Can you recommend any other useful resources?

40.   Please suggest a literature list for my level of knowledge about evaluation in P/CVE.

- Information-seeking prompts: These explored the *whys* and *hows* of the evaluation process.
- Recommendation-seeking prompts aimed to solicit guidance, advice, or resources to resolve a problem. These prompts were guided by phrases such as “can you tell me more about”, “can you recommend”, “what is your opinion about”, “shall I”, and so on.
- Task-specific prompts directly requested Copilot’s assistance in certain actions, such as visualising information, providing examples, or offering suggestions. Phrases used in these prompts included “can you visualise,” “can you help me,” “can you provide,” “I need more examples of...,” and “please suggest.”
- Emotional support-seeking prompts were inputted for sharing the feelings, establishing an empathic (emotional) connection, and receiving comforting or any other emotional support. For this category, we asked for help with the feeling of insecurity or fear.

Secondly, we classified the prompts by the number of entries for receiving satisfying feedback:

- Base prompts, which are single statements or questions posed to Copilot also known as “one-shot prompts” ( Dang *et al.*, 2022), mainly used for information-seeking prompts.
- Chain of thought prompts, also known as “few-shot prompts” (Ibid., Szwoch *et al.*, 2024) in which more context was created for Copilot by questions and queries, which together formed a longer dialogue. These were used for more complex prompts, where a certain type of response was needed.

The discussion here was conducted in a single uninterrupted session (without refreshing the chat). This format was essential for holding an immersive discussion that would conclusively determine whether Copilot could be prompted to provide comprehensive knowledge on key aspects of evaluation and draw upon well-established context.

The key aspects (see Table 1) that we wished Copilot to address were based on the INDEED e-guidebooks ^9^ and the tool ^10^. However, our aim was not to pose as a practitioner who would be familiar with these materials before using the chatbot, but rather to suggest one potential way in which the discussion could unfold.

**Table 1.** Key aspects of evidence-based evaluation in P/CVE.

Aspect	Explanation	Prompt no
Definition of evidence- based evaluation and evidence	Evidence-based evaluation is “a process of planning and implementing evaluations which integrates available external evidence, professional expertise and stakeholder values, preferences and circumstances” ( INDEED D1.2).	1, 13, 14
Methods, biases, quality	The methods and evaluation designs are crucial for evaluating the effectiveness of P/CVE programs. These are applied to identify the structure for evaluation and how to collect and analyse data ( INDEED E-guidebook 1).	2, 3, 4, 5, 6, 10, 12, 29, 30, 31
Types of evaluation	The INDEED E-guidebook 1 states that “[t]here are countless types of evaluations that differ from each other in terms of objectives, methods, timing and scope”. (p. 16 ) The INDEED proposed three types – formative, process, and outcome evaluations – as the most essential not only for planning and conducting evaluations per se but also for designing evidence-based programmes.	7, 8, 9, 10, 11
Internal and external evaluators	INDEED E-guidebook 1 defines internal and external evaluators and suggests the criteria for choosing an evaluator as follows: “[a]n external evaluator is someone who does not have a role in or a significant existing relationship with the initiative. External evaluators are typically consultants or academic researchers. An internal evaluator is someone who is currently part of the initiative or the organisation/ institution responsible for it” (E-guidebook 1, p. 13)	28, 32
Evaluation process and evaluation model	One can find different stages of the evaluation process in different sources. The INDEED E-guidebook 2 defined four: preparation, design, execution, and utilisation. Each stage presupposes several steps, explaining to P/CVE practitioners the organisational hurdles for planning P/CVE evaluations (p. 27–36). Because no previously known evaluation model had been developed, the INDEED project designed one to define the principle and processes of evidence-based evaluation in P/CVE ^12^. We also aimed to learn whether Copilot can generate any models in the freely available version.	15, 16, 16 a, 17, 26, 27, 38,
Theory of change	Both the INDEED e-guidebook and Tool note the importance of the Theory of Change (ToC) for evaluation. The components of the theory were described in the E-guidebook 2, (p. 16), but to approach the ToC, practitioners would need more guidance and practical examples from the P/CVE field.	18, 19, 20
Ethics, data protection and gender in evaluation	The ethical and legal dimensions are essential for evaluation and these were also presented in the E-guidebooks and the tool. Copilot was expected to cover the principles and explain how to incorporate these aspects into evaluations (for example, through indicators, ethical data collection process, monitoring and securing storage of data (see E-guidebooks 1 and 2 and the Tool).	21, 22, 23, 24, 15
Other useful aspects related to the challenges in the field		
Trainings and capacity building	Although these issues were not specifically described in the E-guidebooks, the INDEED gap analysis revealed a lack of trainings on evaluation. Here we sought to determine the reaction of Copilot to this question and what it could suggest.	34, 35, 36, 37, 39, 40
Fear of evaluation	The issue of fear of evaluation also came from the gap analysis and preceding discussions with P/CVE practitioners. It was important to investigate whether a chatbot is capable to respond emotionally and encourage practitioners to take the first steps towards evaluations.	49
Examples	The examples of evaluations and practical procedures are crucial for practitioners. The INDEED E-guidebooks collected many useful examples. It was important to study whether Copilot can refer to any, and how useful these were.	19, 33
Local communities and cultural realities	The value of inclusion of local communities in the evaluation process and tailoring evaluation practise towards cultural sensitivities have been discusses in all INDEED outcomes. Even though the evaluation procedures are somewhat standard, the uniqueness of the process comes through the understanding of local practises.	41–50

In the second stage of our research, we developed 10 scenario-based prompts ^11^ to simulate real-world scenarios in which P/CVE professionals would ask the chatbot for context-specific advice ( Box 2). Our scenario-based prompts were provided as one-shot prompts. They included specific details about each professional context, such as policy, social work, policing, and NGO work, to reflect current realities. Some prompts also accounted for various geographical settings and problem types, drawing from the unique challenges faced by P/CVE practitioners in these areas.

**Box 2. List of prompts for Discussion 2 with Copilot**

41.   I work for the police. We urgently need to evaluate our ad-hoc response as part of a P/CVE case. We have no experience with evaluation, we just know the main lines of it. Our management is very supportive, but we do not know where to start. Our resources are limited, and we also have a lot of sensitive data. Can you provide any recommendations about how we can do the evaluation? Would it be better to engage an internal or external evaluator?

42.   I am running a mentoring programme in Europe that helps people to disengage from violent extremism. It is often difficult to quantify its effect. After the programme, we lose contact with our clients. How can I determine whether my programme works?

43.   I am working for an NGO in France, and we are running a programme that is aiming to help returnees from Syria. The authorities would like us to evaluate the programme and present the results of our evaluation to them. How can we evaluate it? How long might the evolution take? What resources will we need if we start from zero?

44.   Our organisation has just designed a new P/CVE programme that aims to improve young people’s resilience to radicalisation in a neighbourhood. Youngsters present the greatest challenge in this neighbourhood. They do not want to attend school; many are part of youth criminal gangs and have problems with law enforcement. How can we determine whether the programme we have designed will be successful?

45.   Our organisation is running a P/CVE programme. We have collected a lot of data during the data collection process. The data is in electronic and written formats. There is a lot of sensitive and private data. Can we use this data for the evaluation process and how? How and where should we store this data before and after the evaluation?

46.   Our NGO is in Sweden. We are involved in the implementation of a three-year P/CVE project. The project is aimed at facilitating the reintegration of radicalised individuals previously convicted of crime into society. We work with a large number of local actors, and cooperation is challenging. We would like to evaluate our project. What kind of data do we need to perform an evaluation? What shall we start with?

47.   My team is preparing for the evaluation of P/CVE strategy at the national level. The strategy is very complex, and it is based on national and local multi-stakeholder partnerships. We work with around 50 partners. How should we organise our evaluation? How can we make sure that our evaluation is ethical?

48.   Our small NGO in the UK promotes depolarisation and anti-radicalisation through a sport project for the members of the local community. Our members are both males and females, and different activities ae organised for both. We need to evaluate our programme to re-apply for funding. How can we evaluate our programme by considering different genders?

49.   Our external evaluator is currently planning an evaluation of the exit strategy in the field of P/CVE that is being implemented by our team. We feel insecure about sharing our data and working methods. Do we need to share everything? What will help us prepare and plan the evaluation together with the evaluator?

50.   As part of the Horizon project, we would like to evaluate a small-scale pilot project in P/CVE. We have not yet started planning it and would already like to prepare all the colleagues for the future evaluation now. The evaluation will be performed by an external agency. What do we need to prepare at this stage to make the evaluation happen? Is there anything good to know at this stage? How can we design our pilot project so that it will be easier to evaluate? None of us have previous experience with evaluation.

Currently available general resources for P/CVE professionals often fail to offer tailored guidance, neglecting (or avoiding for the reason of complexity) the specific needs of practitioners working in diverse contexts. Our aim was to find out whether Copilot can fill in this gap by providing responses and recommendation that speak specifically to their unique situations.

By combining the results from both stages, our study aimed to gain a deeper understanding of Copilot’s capacity for engaging in meaningful and practical discussions with P/CVE professionals.

At this stage, we reviewed Copilot responses as those of a recommender system, which are usually evaluated based on parameters such as personalised information and tailored recommendations ( Lin & Li, 2024). Our assessment of the chatbot’s performance was guided by three *criteria* and a list of predetermined *analysis questions*. The *criteria* of: 1) accuracy and reliability; 2) relevance and integrity; as well as 3) readability and comprehensibility were deemed most relevant for understanding Copilot’s usability and limitations in this context. These criteria were informed by literature and domain expertise. The analysis questions were meant to better explain the limits of these criteria. It is important to note that while more rigorous testing methodologies exist for exploring each criterion, our aim was not to delve deeply into each category but instead provide a practical overview of Copilot’s potential as a prototype for a P/CVE tool, with the help of analysis questions, and determine whether chatbots are worth further exploration.

### b. Limitations and suggestions for further research

It is essential to acknowledge the limitations of this study and outline the prospective next steps in similar research.

Firstly, our primary objective was *not* to find out whether the chatbot can serve as a fully functional evaluator but rather to explore whether a P/CVE professional can obtain the key information and support necessary to plan and implement their own P/CVE work with evaluation in mind. Our purpose was also *not* to assess the chatbot’s overall performance in the P/CVE field or evaluation, but instead to create the first mapping of its potential as of an AI tool for professionals.

A further limitation concerns access to the chatbot. To explore Copilot, we utilised the open-access version of Copilot without logging in, which can be regarded as a constraint on our study. Accessing the full version, which requires an Office 365 account, would have enabled us to leverage additional functionalities, such as, for instance, image generation or voice communication (in some regions). These are not available in the open-access version but exploring them would broaden the spectrum of use-cases for P/CVE. This is something to be considered in further studies. We also explored LLM chatbots mainly as a knowledge-based recommender system but more studies could be done to explore other functions of chatbots, especially task-specific ones, by using the full access to the system and exploring the new agentic features of the chatbot. To add further to research on the performance of such chatbots in various languages, functions such as usability could be better explored.

There are also several technical limitations worth noting. One key limitation concerns the number of iterations for inputting the prompts. There is an ongoing discussion about the variability of responses from chatbots, which can impact credibility if not managed effectively. In this study we inputted our prompts in one iteration, whereas multiple iterations might be necessary to explore the stability of responses. In 2024, we conducted a similar review of Copilot responses ^13^ in three iterations and did not spot a significant divergence in the answers received. Given the rapidly improving performance of LLMs and chatbots, including their stability, such an evaluation was deemed beyond the scope of this study. Moreover, the differences in performance between various LLMs and chatbots highlight the complexity of assessing these models’ capabilities.

The discussion with Copilot was based on a range of prompts engineered by the authors, aimed at simulating user experience. Prompt engineering refers to the techniques used to guide conversations with LLM-based chatbots to yield effective and relevant responses ( Ekin, 2023; Giray, 2023; White *et al.*, 2023). This study drew upon the authors’ extensive interactions with European and non-European practitioners and policymakers in the field of P/CVE, providing insight into their perspectives on evidence-based evaluation and the needs of the field. It is well established that unlocking the full potential of chatbots and achieving optimal results from queries requires the user to know how to formulate effective prompts. However, not all users possess this knowledge, which can impact the quality of interactions with these systems. Furthermore, involving real practitioners in such a study would have added richness and depth to the research findings. This would be even more so if we were able to detect more nuances in the usage of these systems through studying interactions of different P/CVE professionals with chatbots.

### c. Criteria for analysis

This section outlines the criteria developed for evaluating Copilot responses. Although these criteria may appear to overlap to some degree, we have identified them as distinct and separately explained their borders in Table 2. These criteria were established to provide clarity on how Copilot or similar LLM systems can potentially support P/CVE practitioners by providing knowledge, recommendations, and some other forms of support.

**Table 2.** Analysis questions.

Category	Analysis questions
Accuracy and reliability	1. Are the responses (definitions and concepts) factually correct? 2. If there are inaccuracies, how consequential are they? Can they mislead the professional in significant ways? 3. Is there deviation in responses, for example, unexplained facts from other fields and sectors? 4. Are there references to the relevant literature sources?
Relevance and integrity	1. Do the responses show that Copilot understands what P/CVE and evaluation mean? 2. Do the responses cover key issues related to the topics of P/CVE and evaluation? Does Copilot talk about specifications in P/CVE evaluation, for example, in relation to ethics and sensitivity? 3. How practical and useful is the information provided? Do responses contain recommendations, tips, and know-how, and suggest pathways to evaluation?
Readability and comprehensibility	1. Are things explained in an easily accessible manner? 2. Is there a large number of specific terms that require additional search to understand their meaning? Or is the meaning clear from the context? 3. Is the response logically structured?

***1. Accuracy and reliability.*** There are reports about inaccuracies and lack of reliability of LLM-based chatbots across different fields. Such errors can have significant consequences for users, leading to discussions about accountability for these mistakes. For example, one paper (see Aaronson, 2024) and an earlier BBC article (see Yagoda, 2024) described the repercussions of an error in an airline’s AI chatbot for travelers, and the response from the management was that the chatbot was “responsible for its own actions”. In the human-driven field of P/CVE, such errors could be especially harmful, as they might affect individuals’ lives. Based on previous works, Aaronson (2024) refers to LLM chatbots as “fallible”, meaning that they produce incomplete and often outdated data, not to mention factual errors and even disinformation (p. 2). However, it is important to remain optimistic, noting that LLMs are continuously improving, and there is a wide variety of them with different levels of reliability and accuracy. Several recent medical studies on LLM chatbots have concluded that these systems have great potential for professional use, due to their generally good accuracy ( Benirschke *et al.*, 2024; Chow & Li, 2025; Kamminga *et al.*, 2025; Stroop *et al.*, 2024). Gupta *et al.* (2024) conducted rigorous scientific testing ^14^ of three chatbots – Google Gemini, Microsoft Copilot, and OpenAI ChatGPT – in the medical domain. They found that Copilot demonstrated greater reliability compared to other systems. The study also revealed that Copilot made reference to more scientific sources and had a higher number of citations than Gemini. These criteria have not yet been reviewed in P/CVE research in relation to evaluation, indicating a need for a more structural and focused approach. Therefore, our goal here is to explore these categories through analysis questions and observations, and equally evaluate any potential negative impact from the use of LLMs.

***2. Relevance and integrity.*** Unlike accuracy and reliability, relevance and integrity are typically studied within the first two categories in AI LLM evaluation studies. However, for us, these criteria are meaningful and deserve to be addressed individually, as they allow us to understand whether the content produced is relevant first and foremost to P/CVE as a whole, and, secondly, to P/CVE practitioners. From an epistemological perspective, we are eager to explore whether Copilot answers include diverse segments of knowledge that contribute to the overall picture of P/CVE evaluation. In this regard, we acknowledge that relevance could be classified as both *contextual*, referring to the connection between different parts of responses related to P/CVE, and *practical*, meaning the applied and hands-on value of the information to professionals in correspondence with the purpose of the query. We will focus especially on the key aspects outlined in Table 1 above. Several prompts were specifically designed to examine these knowledge segments.

***3. Readability and comprehensiveness.*** A comprehensive understanding of the text is primary associated with its readability. The Flesch-Kincaid Grade Level is one qualitative tool that measures the readability level of texts, aiming to ensure they are accessible to at least college students or graduates (see Gupta *et al.*, 2024). There are many other tests designed to evaluate text readability, particularly in educational contexts (see eg., Nikolayeva, 2024). Readability depends on a system’s ability to introduce and explain terminology effectively. This includes insuring that the content is clear and comprehensive for its intended audience. Unlike students, P/CVE stakeholders also operate within specialised language that includes both organisational and professional jargon, terminology, and a set of acronyms specific to different contexts such as intelligence, policymaking, not to mention prison or social work. Copilot is expected to produce texts that are readable by all these professionals and should explain new terms from the evaluation sector clearly. Additionally, the system should provide pathways for further materials, such as literature sources, or suggest additional questions to improve understanding without relying on traditional web searches. Structure enhances the comprehensibility of the text by breaking down complex content into more digestible chunks, thereby impacting readability. It is important to explore how well Copilot responses are structured and whether recommendations can be extracted easily from these responses.

To explore Copilot’s performance as a recommender system in addressing these issues, a list of detailed analysis questions was prepared for both parts of the study (see Table 2). The analysis questions are qualitative and open-ended. They were designed to lead to a more detailed understanding by identifying specific aspects or patterns in Copilot’s responses. The findings will be described in the next chapter and will be based on these questions. At this stage, it is most important to understand what the limits of Copilot’s capabilities and knowledge are and how well Copilot can understand P/CVE evaluation-related prompts. 

### Findings

The section below will outline our findings based on our review of Copilot responses. To structure this section, we broke it down by previously defined criteria for analysis and the analysis questions.

***1. Accuracy and reliability.*** The prompts designed for Copilot were targeted to find out whether key topics and definitions from the field of evaluation in P/CVE are accurate enough and make sense for practitioners based on the observations of domain knowledge experts. The findings showed that Copilot can lead professional conversations about evaluation, and the information provided is mostly factually correct. The explanation of terminology was generally accurate. One-shot prompts might work as a good starting point to initiate discussions and provide quick understanding of specific terms. For example, when asked to define evidence-based evaluation, the chatbot surprisingly referred to the definition established by the INDEED project based on literature analysis ^15^. This was particularly important because evidence-based evaluation is relevant in many fields such as development, crime prevention, or policy work, where other, more specific to the field, definitions might be used. Moreover, Copilot managed to maintain a clear link between evaluation practises and P/CVE in almost all responses. Second, when Copilot was asked about the differences between various terms that are often confused, Copilot provided sharp distinctions without ambiguity. For instance, Copilot effectively explained the difference between impact evaluation and impact assessment and clarified the distinction between outcome evaluation and impact evaluation (Prompt no. 8), which can somewhat puzzle those without prior knowledge. Additionally, the definition of evidence was satisfying enough; first, Copilot provided a general understanding of evidence, followed by a contextual explanation (Prompt no. 13). 

Beside definitions, we asked Copilot if it could recommend any professional trainings (Prompt no. 35). Out of all the prompts, the answers about the training were least persuasive and accurate because they referred to general trainings, conferences and workshops. This is unsurprising given that there are relatively few specialised trainings on evaluation available, and information about them might not be always publicly accessible. Similar conclusions can be drawn about the examples of existing evaluation from P/CVE (Prompt no. 33). Although Copilot provided a response, it listed evaluation frameworks, useful resources, and guides instead. This indicated a certain degree of inaccuracy, but at the same time echoed the well-established fact that there are yet not many (publicly available) evaluations in P/CVE.

There were instances when the answers looked somewhat imprecise, with concepts and ideas being mixed. For example, participatory evaluation was listed as an evaluation method (Prompt 2), whereas it is instead an approach. Another case involved cost-benefit analysis being listed both as a method and a type of evaluation in responses to prompts no. 2 and 7. This might be confusing when trying to understand how and in which situations it can be applied. Nevertheless, these details are relatively minor inaccuracies that might also go unnoticed by practitioners given the overall accurate and straightforward performance of the chatbot. Additionally, Copilot’s answers in these cases were not necessarily misleading; they simply required more precise classification.

In our simulation case, where we posed as practitioners with limited knowledge of the filed, we explored whether an understanding of evaluation could be built up gradually through a series of prompts. The chain of thought prompt provided a clear pathway to key knowledge and opened up additional pathways for further exploitation within each suggested category. It is worth noting that in older versions of Copilot (2023–2024) the chatbot in the open-access version could suggest follow-up questions to continue and drive the discussion, which is no longer the case in the latest version. Such follow-up questions could be instrumental for newcomers to the topic but might also stifle creative and active thinking during learning, potentially turning users into passive learners. This limitation would probably restrict Copilot’s ability to truly “learn” from user behaviour and predict preferences and interests.

Finally, the original answers sometimes lacked references and sources. However, users can request references as part of their prompts but not retroactively. The sources offered by Copilot were based on a mix of American and European open-source materials available online. There is still room for more precise quotations and references that can be easily accessed within the same webpage rather than requiring users to visit external websites.

***2. Relevance and integrity.*** Our analysis of relevance and integrity includes the study of responses from all 50 pre-designed prompts. By carefully reviewing the answers, we established that Copilot is capable of leading conversations about basic topics related to evidence-based evaluation in the field P/CVE.

As previously noted, establishing a more thematically rich dialogue with Copilot requires a session where all prompts are produced sequentially. If all the prompts are generated within one session, Copilot readily captures the thematic context, behavioural preferences, and interests of the user, providing an immersive and engaging dialogue within the topic limits.

We also received promising results from scenario-based prompts, where the context is described directly in the prompt rather than extracted from the ongoing dialogue. Therefore, both techniques—sequential prompting and scenario-based prompting—are effective for obtaining meaningful recommendations from Copilot.

All key aspects of evidence-based P/CVE evaluation from Table 1 were included in the prompts. Copilot’s responses in relation to relevance and integrity were generally much better than satisfactory. For instance, by prompts no. 22 and 22, which asked about gender and ethics without directly mentioning evaluation or P/CVE (presumably because the context was already established), we received valuable information. The response to prompt 22 provided insights on the importance of considering gender in P/CVE evaluations, offered relevant recommendations, included practical examples for gender-sensitive evaluations, and supplied external links to sources. Similarly, in prompt no. 23, Copilot emphasised the significance of human rights in evaluation, gave hands-on guidance on conducting a human-rights-based evaluation, and directed us to additional resources. As a result, through the detailed knowledge and guiding instructions provided by Copilot, practitioners can access almost all the details necessary for evidence-based evaluations, enriched with (mostly) valuable examples and supported by external sources.

Copilot demonstrated its great potential as a recommender system by proving hands-on advice and recommendations in both one-shot and few-shot prompts. When we asked for practical assistance to get started with various aspects of evaluation (since starting can often be challenging), Copilot offered concrete tips on initial and subsequent steps. In prompts no. 12, 15, 18, and 43, Copilot provided detailed step-by-step instructions. Each set of recommendations began by setting up relevant objectives before moving on to further steps.

Copilot was also helpful in guiding us through options. For instance, in scenario prompt no. 41, we asked for recommendations on whether to choose an internal or external evaluator for a police case evaluation. Copilot recognised the sensitivity of this context and recommended engaging an internal evaluator. The recommendation was highlighted in bold letters to emphasise Copilot’s preferred option. While such recommendations are valuable, it is still crucial to weigh all pros and cons independently before making a final decision, ensuring personal accountability.

In session 1, during our extensive conversation, Copilot demonstrated a clear understanding of our interests and could predict our subsequent prompts somewhat. For example, when we asked about the Theory of Change (Prompt no. 18), Copilot provided an example relevant to P/CVE, anticipating our next question (prepared in Prompt 19). This ability to anticipate future needs showcased Copilot’s effectiveness as a conversational assistant.

Scenario-based single-shot prompts demonstrated that clearer communication with Copilot can lead to better results. The effectiveness of communication largely depends on practitioners’ preparedness and awareness of various aspects of P/CVE. In scenario prompt no. 42 we posed as a practitioner who was unfamiliar with P/CVE but operated in a disengagement programme and wanted to understand how to assess whether the programme was effective. Without directly using phrases like “P/CVE” or “evaluation”, our goal was to see if Copilot would discuss evaluation concepts. Contrary to expectations, Copilot did not explicitly mention “evaluation” but instead focused on specific forms of it, such as impact assessment and self-assessment. In this context, a real well-informed evaluation expert might recommend referring the practitioner to process evaluations or formative evaluations, which would be more relevant given the scenario. At the same time, self-assessment remains a good starting point for practitioners to begin thinking about some evaluation strategies.

Understanding how relevant and integral Copilot’s responses are under complex scenarios was one of the main intentions of this study. In the second session, each prompt contained a significant amount of information delivered in a single shot without building pre-context through prior prompts. We wondered whether Copilot could orient itself effectively to the initial data and respond comprehensively to all the details provided. We were particularly interested in how well Copilot would incorporate specific details such as geographical data or programme focus into its responses, producing more tailored and context-specific answers. As a result, we discovered that geographical details in our prompts (Prompts no. 42, 43, 46, and 48) were not consistently taken up to produce more tailored responses. On the other hand, Copilot provided more contextualised information when it was given specific organisational specialisations, such as working with returnees or young people. This suggests that while Copilot can handle complex prompts, not all the details provided can lead to a more precise and tailored answer.

Surprisingly, Copilot acted as a rather emotional, supportive, and understanding mentor throughout our interactions. For example, in prompt no. 49, the scenario described the fear of evaluations and insecurities about external evaluators. In response, Copilot was empathetic but also very encouraging, offering practical advice to help overcome these fears and resolve the issue constructively. Copilot provided reassurance while emphasising the importance of preparing the team for the evaluation process. This included suggesting ways to familiarise the team with the evaluation methods and objectives, ensuring transparency and open communication, and providing training or workshops to build confidence and competence. By doing so, Copilot helped create a supportive environment that encouraged the team to face their fears head-on.

***3. Readability and comprehensibility.*** We all know how overwhelming it can be to absorb new information. The language used to explain this information, along with visual aids, plays a crucial role in learning as they affect both readability and comprehensibility. We explored these criteria primarily through the first 40 prompts in Session 1 and several scenario-based prompts in Session 2. To investigate these criteria, we repeatedly informed Copilot that we were practitioners who lacked extensive knowledge about evaluation and sought guidance and assistance.

**Figure 1.**
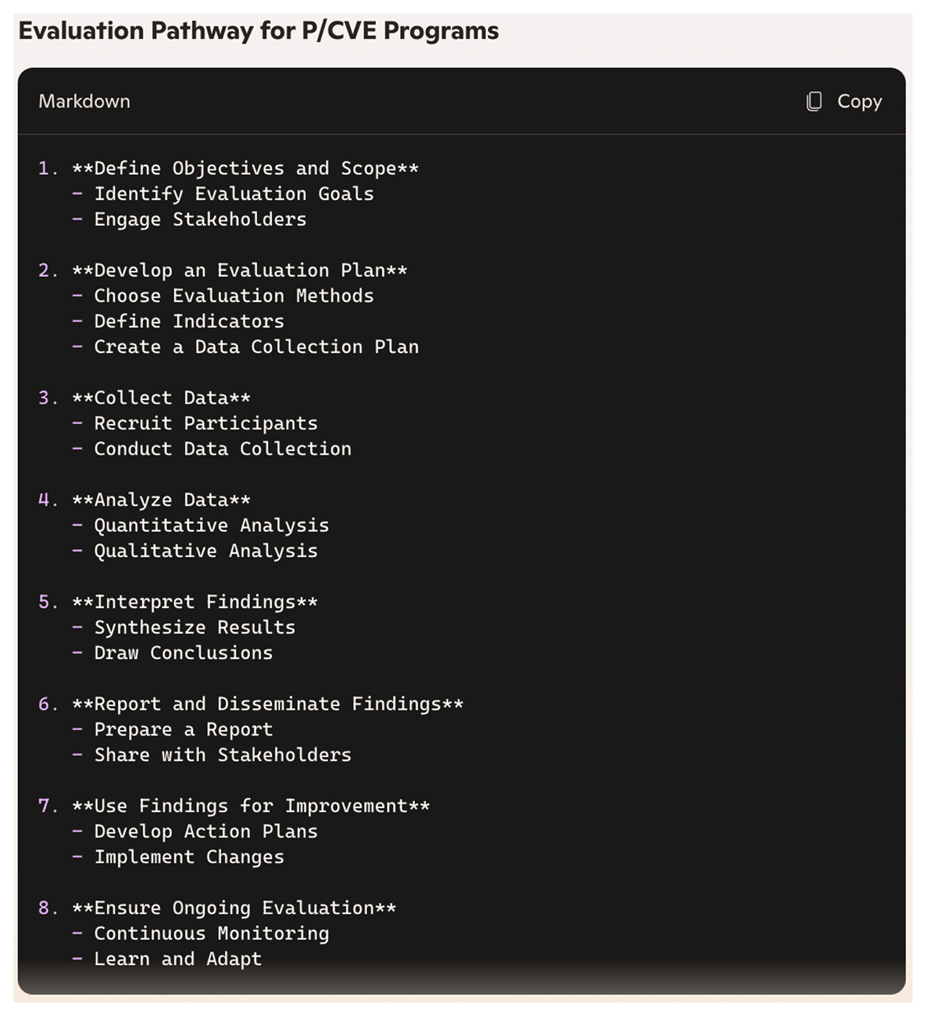
Visualisation produced by Copilot in prompt no. 16 (screenshot).

**Figure 2.**
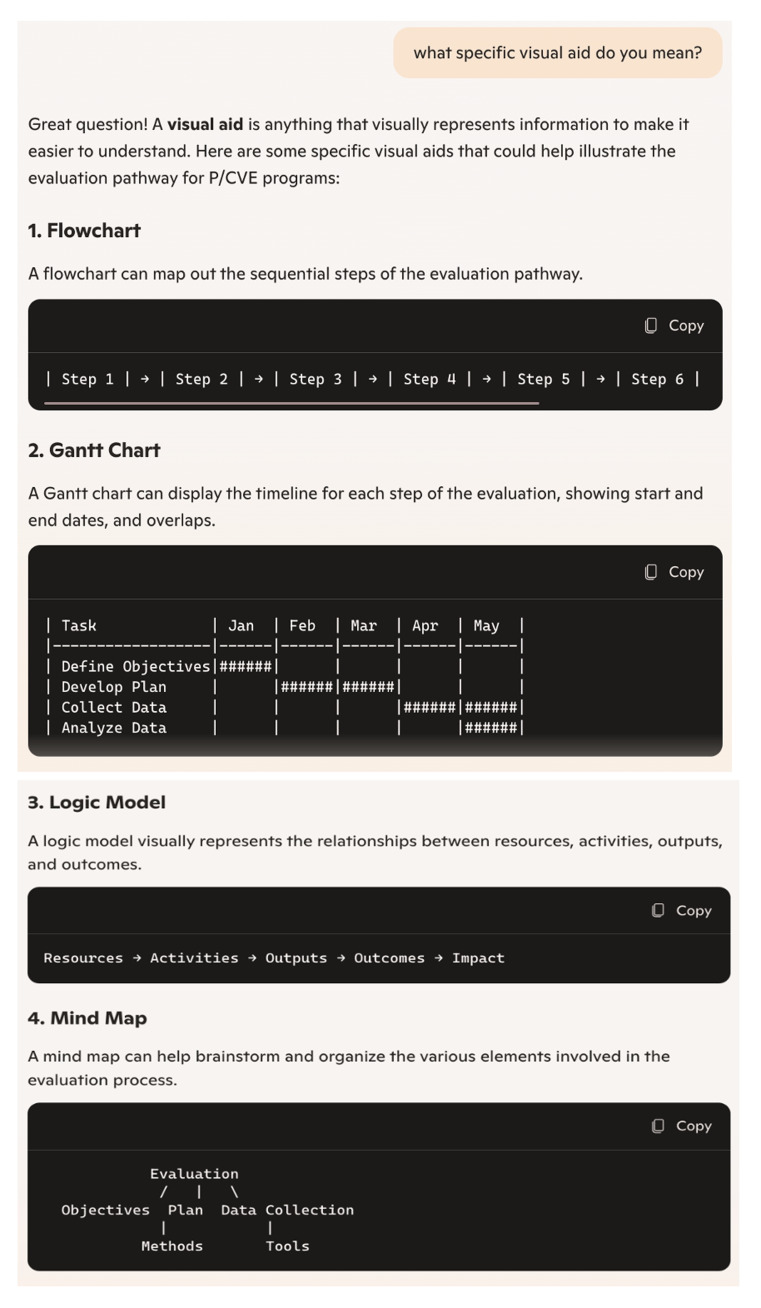
Visualisation produced by Copilot in prompt no. 16a (screenshot).

During the first session, Copilot effectively gauged our level of knowledge through a number of prompts. To enhance readability and comprehensibility, Copilot employed several techniques to adapt its responses to our level of expertise. Firstly, as we had already concluded during the analysis of relevance, Copilot excelled in breaking down complex queries into simpler parts and addressing each part separately for better comprehension. This approach was particularly evident when we explicitly positioned ourselves as novices in evaluation. Secondly, Copilot utilised diverse visual aids that effectively broke down content into more digestible chunks (See Figure 1 and Figure 2). Although the version of Copilot we used (without sign-in) produced graphs with limited creativity and color, given the current capabilities of similar systems, we believe Copilot could offer more impactful and creative visualisations. Notably, Copilot continued to provide these visual aids throughout the session without us directly requesting them. Thirdly, at the end of some prompts, Copilot proactively summarised responses or proposed checklists, making it easier for us to retain all key points.

These methods, such as breaking down complex information into simpler parts, providing diverse visual aids, and summarising responses, could be instrumental in developing tailored processes based on individual needs and culture. They lay a strong foundation for initiating evaluation efforts.

## Conclusion

In contrast to widely discussed research on the risks associated with AI LLMs in addressing P/CVE, this study highlights the instrumental value of using LLMs for P/CVE efforts. We examined Copilot, a publicly available chatbot that combines an LLM with recommender system capabilities, as a practical tool to enhance evidence-based policy and practice in the field. The study involved a literature review and analysis of Copilot’s responses to 50 pre-designed prompts. This analysis provided preliminary conclusions about the practical value of using Copilot. Although the study had certain limitations and could benefit from more rigorous multi-method approaches, it demonstrated that Copilot can serve primarily as an educational tool for P/CVE practitioners. Crucially, Copilot can also function as a companion and mentor in P/CVE work by offering emotional support through natural language dialogues. By engaging users in meaningful conversations, Copilot can inspire and encourage them to acquire new knowledge about the subject matter. This approach can help overcome barriers such as fear of the unknown and foster positive planning and utilisation of new information within organisations.

In this study, we investigated how useful Copilot can be in understanding complex processes, models, and approaches related to planning and conducting evaluations. Our findings demonstrated that chatbots like Copilot can provide comprehensive knowledge on key aspects of evaluation, including definitions, evaluation types, methods, and processes. Copilot was particularly effective in demonstrating and visualising evaluation pathways and models, as well as providing ideas for initial steps towards evaluations. The question remains, however, whether Copilot’s responses alone are sufficient to kick-start the process effectively in practise. The chatbot also provided relevant literature and additional sources that can significantly deepen practitioners’ knowledge. Nonetheless, it is crucial to ensure that these sources are systematically selected and rigorously checked for accuracy and currency, so they can be reliably consulted by P/CVE professionals.

At this stage, it is essential to address concerns around accuracy and reliability, which may be influenced by the quality and diversity of training data. One major weakness is the trustworthiness and accuracy of all the bits of information provided by Copilot that still require professional knowledge and expertise to filter responses. Evidently, it is still important to keep not only a human-in-the-loop but also a professional-in-the-loop to verify the accuracy of the arguments in suggested responses. To increase the value of such tools, incorporating more diverse and high-quality training data specific to the P/CVE field would be crucial. Implementing fact-checking mechanisms to ensure the accuracy of information provided is another way to enhance its performance and reliability.

According to our review, the pros of this solution still outweigh the cons. The strongest aspect of Copilot was its ability to break complex content down into smaller, more digestible pieces, helping practitioners navigate through the sea of information without having to spend time searching, selecting, and reviewing different resources on the Internet in the traditional way. This capability might be especially promising for optimising practice, making it easier and more efficient for practitioners to access and utilise relevant information. The voice function that is already available in some models can make this experience even more natural and effective.

The overall development of LLMs is expected to continue influencing the performance of chatbots like Copilot, enhancing their accuracy and relevance over time. As these models improve, they will become increasingly suitable for multipurpose professional use, including in the P/CVE field. However, it remains crucial to develop and apply user-centred approaches to critically assess their output when considering the use of such tools to inform professionals. Pondering both the potential and limitations of using chatbots like Copilot for evidence-based evaluation in the P/CVE field, LLM-based chatbots are worth further exploration to identify more opportunities that this technology can bring to other types of P/CVE work. Regular reviews should be conducted to identify new opportunities related to the rapid development of LLMs and their applications in the P/CVE field.

## Ethics and consent

Ethical approval and consent were not required

## Data Availability

Zenodo: Copilot's responses, January 2025. https://doi.org/10.5281/zenodo.14869752 [ Van der Vet & Malkki (2025). Copilot's responses, January 2025 [Data set]. Zenodo: Copilot's responses, March and April 2024. https://doi.org/10.5281/zenodo.14814720 [ Van der Vet & Malkki (2025). Copilot's responses, March and April 2024 [Data set]. Data are available under Both: the terms of the Creative Commons Zero "No rights reserved" data waiver (CC0 1.0 Public domain dedication). the terms of the Creative Commons Attribution 4.0 International license (CC-BY 4.0).
