# Supplementary material for: Copilot in service: Exploring the potential of the large language model-based chatbots for fostering evaluation culture in preventing and countering violent extremism
Source: Open Res Eur. 2025 Apr 17;5:65. Originally published 2025 Mar 7. [Version 2] doi: 10.12688/openreseurope.19612.2 (PMC12498320; doi:10.12688/openreseurope.19612.2)
Supplement: Peer review history

# Reviewer report: Reviewer response for version 2

**DOI:** 10.21956/openreseurope.21801.r53273

**Published:** 2025-05-08

**Authors:** Krithi Pushpanathan

**Affiliations:** 1 National University of Singapore, Singapore, Singapore

No further comments, thank you.

If applicable, is the statistical analysis and its interpretation appropriate?

Not applicable

Is the study design appropriate and is the work technically sound?

Yes

Is the work clearly and accurately presented and does it engage with the current literature?

Yes

Are the conclusions drawn adequately supported by the results?

Yes

Are sufficient details of methods and analysis provided to allow replication by others?

Partly

Are all the source data and materials underlying the results available?

Partly

Reviewer Expertise:

AI, LLM, Ophthalmology

I confirm that I have read this submission and believe that I have an appropriate level of expertise to confirm that it is of an acceptable scientific standard.

# Reviewer report: Reviewer response for version 2

**DOI:** 10.21956/openreseurope.21801.r53274

**Published:** 2025-04-26

**Authors:** James C L Chow

**Affiliations:** 1 University Health Network, Toronto, Ontario, Canada

The authors have addressed all my concerns.

If applicable, is the statistical analysis and its interpretation appropriate?

Yes

Is the study design appropriate and is the work technically sound?

Yes

Is the work clearly and accurately presented and does it engage with the current literature?

Yes

Are the conclusions drawn adequately supported by the results?

Yes

Are sufficient details of methods and analysis provided to allow replication by others?

Yes

Are all the source data and materials underlying the results available?

Yes

Reviewer Expertise:

AI, LLM, ML, Chatbot, Radiotherapy, Computer simulation.

I confirm that I have read this submission and believe that I have an appropriate level of expertise to confirm that it is of an acceptable scientific standard.

# Reviewer report: Reviewer response for version 1

**DOI:** 10.21956/openreseurope.21215.r51988

**Published:** 2025-03-25

**Authors:** Krithi Pushpanathan

**Affiliations:** 1 National University of Singapore, Singapore, Singapore

This manuscript makes a significant and timely contribution to the ongoing discourse on utilizing large language models (LLMs) for preventing and countering violent extremism (P/CVE). Through the qualitative analysis of its responses to 50 pre-designed prompts, this study evaluates Copilot as a recommender system, providing valuable insights into its potential to enhance evidence-based policy and practice within the P/CVE field. Overall, the manuscript is well-written and contributes meaningfully to the ongoing conversation on AI-assisted decision-making in P/CVE. That said, I have a few minor suggestions that could further refine the manuscript:

1. The authors assert that "limitations such as ‘bullshitting’ and hallucinations in early AI developments (Zhao et al., 2023) are no longer a primary concern." However, recent research (e.g., Steyvers et al., 2025) continues to demonstrate that hallucinations remain a significant issue and that users tend to overestimate the accuracy of LLM-generated responses. Given these findings, I would caution against the claim that hallucinations are no longer a primary concern. (see: Steyvers, M., Tejeda, H., Kumar, A. *et al.* What large language models know and what people think they know. *Nat Mach Intell* **7**, 221–231 (2025). https://doi.org/10.1038/s42256-024-00976-7)
2. Section C in Methods, Criteria for Analysis, provides a clear overview of the evaluation framework and methodology. However, it does not specify who conducted the qualitative assessment of Copilot’s responses. Were these evaluations performed by domain experts, researchers, or practitioners in P/CVE? Additionally, how many evaluators were involved? If multiple evaluators participated, were any measures taken to ensure consistency, such as inter-rater reliability assessments? Providing this information would enhance the transparency and credibility of the evaluation process.
3. The Findings section presents key results, but given that LLMs evolve continuously through manual updates and user interactions, it is unlikely that identical responses will be obtained in future replications of this study. To facilitate longitudinal research and ensure transparency, I strongly recommend that the authors provide the full set of raw data (i.e., Copilot’s responses alongside the corresponding evaluations) as a supplementary table. This would allow future researchers to assess improvements in LLM performance over time and strengthen the reproducibility of the study’s findings.

If applicable, is the statistical analysis and its interpretation appropriate?

Not applicable

Is the study design appropriate and is the work technically sound?

Yes

Is the work clearly and accurately presented and does it engage with the current literature?

Yes

Are the conclusions drawn adequately supported by the results?

Yes

Are sufficient details of methods and analysis provided to allow replication by others?

Partly

Are all the source data and materials underlying the results available?

Partly

Reviewer Expertise:

AI, LLM, Ophthalmology

I confirm that I have read this submission and believe that I have an appropriate level of expertise to confirm that it is of an acceptable scientific standard, however I have significant reservations, as outlined above.

## References

[1] : What large language models know and what people think they know. *Nature Machine Intelligence* .2025;7(2) : 10.1038/s42256-024-00976-7 221-231 10.1038/s42256-024-00976-7

# Response

**Published:** 2025-04-02

**Authors:** Irina van der Vet

Dear Krithi Pushpanathan, Thank you very much for your review; it was very helpful. Below please find our responses to your points, where we have done our best to address each one.

1. “The authors assert that "limitations such as ‘bullshitting’ and hallucinations in early AI developments (Zhao et al., 2023) are no longer a primary concern." However, recent research (e.g., Steyvers et al., 2025) continues to demonstrate that hallucinations remain a significant issue and that users tend to overestimate the accuracy of LLM-generated responses. Given these findings, I would caution against the claim that hallucinations are no longer a primary concern. (see: Steyvers, M., Tejeda, H., Kumar, A. et al. What large language models know and what people think they know. Nat Mach Intell **7**, 221–231 (2025). https://doi.org/10.1038/s42256-024-00976-7)”

***Author Response: **Thank you for this comment. By stating that hallucinations are no longer a primary concern, we meant to suggest that efforts have been made to manage this issue since the introduction of early chatbots, using various measures. To highlight this point better, we changed the wording and added an additional reference. At the same time, we agree with you that accuracy issues still persist, even with these measures in place. We followed your recommendation and revised the statement to provide more explanation. Please kindly see if our changes in the text reflect your point.*

1. “Section C in Methods, Criteria for Analysis, provides a clear overview of the evaluation framework and methodology. However, it does not specify who conducted the qualitative assessment of Copilot’s responses. Were these evaluations performed by domain experts, researchers, or practitioners in P/CVE? Additionally, how many evaluators were involved? If multiple evaluators participated, were any measures taken to ensure consistency, such as inter-rater reliability assessments? Providing this information would enhance the transparency and credibility of the evaluation process”.

***Author Response: **Thank you very much for this useful comment. Following your recommendation, in section A, we clarified that we are domain experts (evaluation in P/CVE) - please see whether this brings more clarity. In addition, it was specified already in the earlier version that: “This study drew upon the authors’ extensive interactions with European and non-European practitioners and policymakers in the field of P/CVE, providing insight into their perspectives on evidence-based evaluation and the needs of the field.” Our methodology was based on our earlier publications on evaluation in P/CVE, which also demonstrates our expertise. From the beginning of this paper, we have been careful not to call our study an “evaluation” but rather referred to it as “mapping” and “review.” This is important because our aim was not to conduct a multidimensional evaluation, which would require a more holistic approach with experts from different domains. Instead, our goal was to provide a snapshot of existing research possibilities and share our initial observations for our taregt audience: practitioners, policymaker and researchers in P/CVE.*

1. “The Findings section presents key results, but given that LLMs evolve continuously through manual updates and user interactions, it is unlikely that identical responses will be obtained in future replications of this study. To facilitate longitudinal research and ensure transparency, I strongly recommend that the authors provide the full set of raw data (i.e., Copilot’s responses alongside the corresponding evaluations) as a supplementary table. This would allow future researchers to assess improvements in LLM performance over time and strengthen the reproducibility of the study’s findings”.

***Author Response: *** Thank you for this comment. Indeed, providing the full datasets aligns with sustainable research practices. As per the Open Research Europe requirements, we initially have made our full datasets (prompts + the chatbots’ responses) available in open access to support the principle of reproducibility. These datasets were mentioned in the paper and listed in the references section. *Since we referred to these published datasets, we found it redundant to duplicate them inside our paper. Please see our datasets here:*

- Van der Vet I, Malkki L: Copilot's responses, January 2025. Zenodo. [Data set].2025. http://www.doi.org/10.5281/zenodo.14869752

- Van der Vet I, Malkki L: Copilot's responses, March and April 2024. Zenodo. [Data set].2025. http://www.doi.org/10.5281/zenodo.14814720

Thank you again for your review, Authors

# Reviewer report: Reviewer response for version 1

**DOI:** 10.21956/openreseurope.21215.r51991

**Published:** 2025-03-12

**Authors:** James C L Chow

**Affiliations:** 1 University Health Network, Toronto, Ontario, Canada

This study evaluated the effectiveness of an LLM-based Copilot chatbot in preventing and countering violent extremism. After reviewing the work, I found it both topical and timely. However, I have a few minor concerns:

1. The authors should include a brief introduction to AI, NLP, and LLM at the beginning of the Introduction section.
2. The authors need to justify why they chose to test the Copilot chatbot instead of other popular bots such as Gemini and ChatGPT. Additionally, they should specify the version of Copilot used in this study.
3. When discussing the medical applications of LLM-based chatbots, please reference more recent studies, such as Chow et al. (JMIR Cancer, 2025, 11(1), e66633 and JMIR Bioinformatics and Biotechnology, 2024, 5, e64406).
4. In the discussion of ethical issues related to the chatbot, the authors should include important references such as Chow et al. (Front Artif Intell, 2023, 6, 1166014).
5. Before the conclusion, there should be a section on limitations and future prospects, highlighting the limitations of this evaluation study and suggesting areas for future research.

If applicable, is the statistical analysis and its interpretation appropriate?

Yes

Is the study design appropriate and is the work technically sound?

Yes

Is the work clearly and accurately presented and does it engage with the current literature?

Yes

Are the conclusions drawn adequately supported by the results?

Yes

Are sufficient details of methods and analysis provided to allow replication by others?

Yes

Are all the source data and materials underlying the results available?

Yes

Reviewer Expertise:

AI, LLM, ML, Chatbot, Radiotherapy, Computer simulation.

I confirm that I have read this submission and believe that I have an appropriate level of expertise to confirm that it is of an acceptable scientific standard.

# Response

**Published:** 2025-04-02

**Authors:** Irina van der Vet

Dear James C L Chow, Thank you very much for reviewing our paper and suggesting improvements, as well as for sharing valuable resources. Please find below our responses to the points raised:

 1. “The authors should include a brief introduction to AI, NLP, and LLM at the beginning of the Introduction section”. *Following your recommendation, we made revisions to the Introduction section.*

 2. “The authors need to justify why they chose to test the Copilot chatbot instead of other popular bots such as Gemini and ChatGPT. Additionally, they should specify the version of Copilot used in this study”. We explained our choice of Copilot at the beginning of the Method section. Since the versions of the chatbot constantly evolve, we provided references to the specific date when Copilot was used for data collection, indicating the exact version employed. We also referred to the datasets by mentioning the dates of data collection, which correspond with the version of Copilot used. Please kindly find the introduced changes.        

 3. “When discussing the medical applications of LLM-based chatbots, please reference more recent studies, such as Chow et al. (JMIR Cancer, 2025, 11(1), e66633 and JMIR Bioinformatics and Biotechnology, 2024, 5, e64406”). *Thank you for the suggestion! We included the reference.*

 4. “In the discussion of ethical issues related to the chatbot, the authors should include important references such as Chow et al. (Front Artif Intell, 2023, 6, 1166014)”. *Thank you for the suggestion! We included the reference.*

 5. “Before the conclusion, there should be a section on limitations and future prospects, highlighting the limitations of this evaluation study and suggesting areas for future research”. Thank you for the comment. We have incorporated these sections as part of the Method section to better emphasise the connections between the chosen methodology, its limitations, and future research.

 Sincerely, Authors
